# Antigenic variation in African trypanosomes

**DOI:** 10.1016/j.molbiopara.2014.05.001

**Published:** 2014-07

**Authors:** David Horn

**Affiliations:** Division of Biological Chemistry & Drug Discovery, College of Life Sciences, University of Dundee, Dow Street, Dundee DD1 5EH, UK

**Keywords:** Immune evasion, *In situ*, Monoallelic, Polycistronic, Silencing, *Trans*-splicing

## Abstract

Studies on Variant Surface Glycoproteins (VSGs) and antigenic variation in the African trypanosome, *Trypanosoma brucei*, have yielded a remarkable range of novel and important insights. The features first identified in *T. brucei* extend from unique to conserved-among-trypanosomatids to conserved-among-eukaryotes. Consequently, much of what we now know about trypanosomatid biology and much of the technology available has its origin in studies related to VSGs. *T. brucei* is now probably the most advanced early branched eukaryote in terms of experimental tractability and can be approached as a pathogen, as a model for studies on fundamental processes, as a model for studies on eukaryotic evolution or often all of the above. In terms of antigenic variation itself, substantial progress has been made in understanding the expression and switching of the VSG coat, while outstanding questions continue to stimulate innovative new approaches. There are large numbers of *VSG* genes in the genome but only one is expressed at a time, always immediately adjacent to a telomere. DNA repair processes allow a new *VSG* to be copied into the single transcribed locus. A coordinated transcriptional switch can also allow a new *VSG* gene to be activated without any detectable change in the DNA sequence, thereby maintaining singular expression, also known as allelic exclusion. I review the story behind VSGs; the genes, their expression and switching, their central role in *T. brucei* virulence, the discoveries that emerged along the way and the persistent questions relating to allelic exclusion in particular.

## Highlights

• Much of what we know about trypanosomatid biology has its origin in studies on VSGs.
• Monotelomeric *VSG* expression and epigenetic switching are remarkable examples of allelic exclusion.
• DNA repair processes allow a new *VSG* to be copied into the single transcribed locus.

## Graphical abstract

**Figure:**
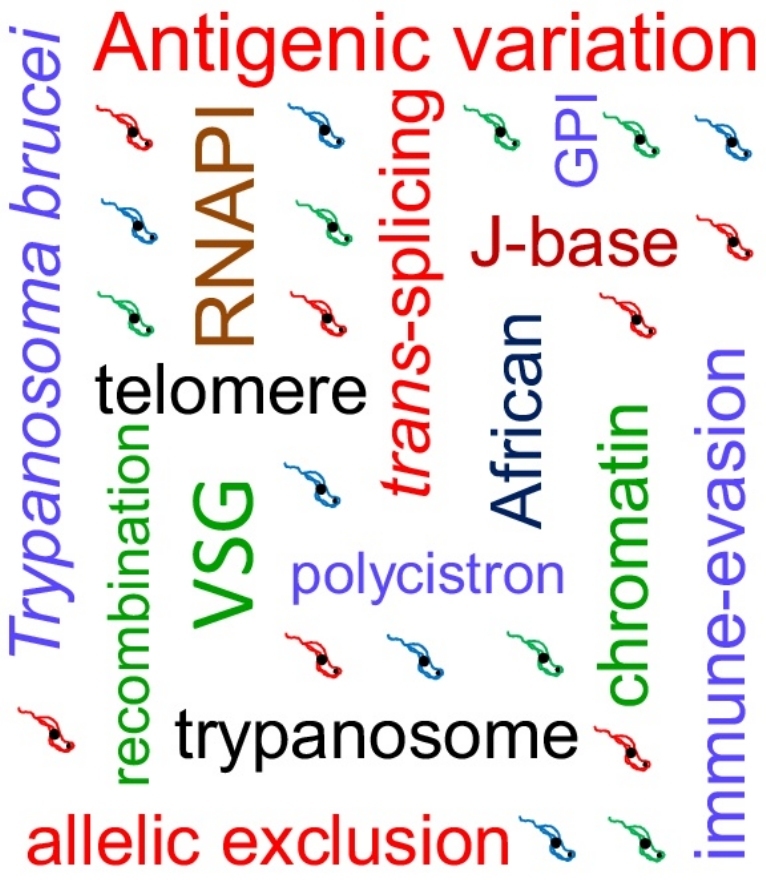

## 1 Introduction

Variant Surface Glycoproteins (*VSGs*) are developmentally regulated genes that mediate immune evasion. Activated in the tsetse-fly salivary-gland [1], and inactivated upon return to the tsetse-fly mid-gut, they produce a protective cell coat throughout the mammalian infectious cycle. The coat must provide robust protection as *Trypanosoma brucei* occupy the bloodstream and tissue-spaces of their hosts and are fully exposed to immune surveillance in this hostile environment. Indeed, as an infection persists, the vast majority of the parasite population is periodically eliminated. The key features underlying successful immune evasion are clone-specific singular VSG expression combined with switching from one VSG to another. The metacyclic cells in the salivary gland are challenging to study since *VSG* expression is heterogeneous during this phase and the yield of *T. brucei* from flies is limiting. Most studies, therefore, have been conducted using bloodstream forms, more recently in axenic culture. Antigenic variation continues to operate in this environment [2] indicating that host antibodies are selective rather than a trigger for variation. An advantage here is that *VSG* switching operates at a frequency of approximately 1 switch/10^5^ cells per population doubling, allowing the analysis of almost homogeneous but switchable populations.

Many seminal discoveries have emerged from studies on VSGs in *T. brucei* and the drive to understand VSGs and their expression has also led to the development of many of the tools and technologies now available for a range of other studies on trypanosomatids. Indeed, studies on *VSG* expression revealed much of what we now know about gene expression in trypanosomatids. Some features are specific to *VSG* gene expression sites, while others operate across the genome and are conserved in trypanosomatids that do not express *VSGs*. Thus, work on VSGs has informed studies on other important parasites, including *Trypanosoma cruzi* and *Leishmania* species. *VSGs* in *T. b. gambiense*, *T. b. rhodesiense*, *T. equiperdum*, *T. congolense* and *T. vivax* are not discussed in detail here but a similar system of gene expression and antigenic variation appears to operate in *T. brucei brucei* and in these other African trypanosomes.

What I present below is a somewhat historical perspective on antigenic variation in *T. brucei* and, in this regard, I recommend further reading of some of the older papers in particular, not often cited these days but often impressive when viewed in this historical context. It is also worth noting that few studies on antigenic variation in *T. brucei* have been or are currently specifically focussed on the prospect of a therapy in the short term. The central role of VSGs in virulence does mean that improved knowledge in this area is likely to present further opportunities for intervention, however.

## 2 A very brief early history – pre *VSG* gene-cloning

**Fig. 1:**
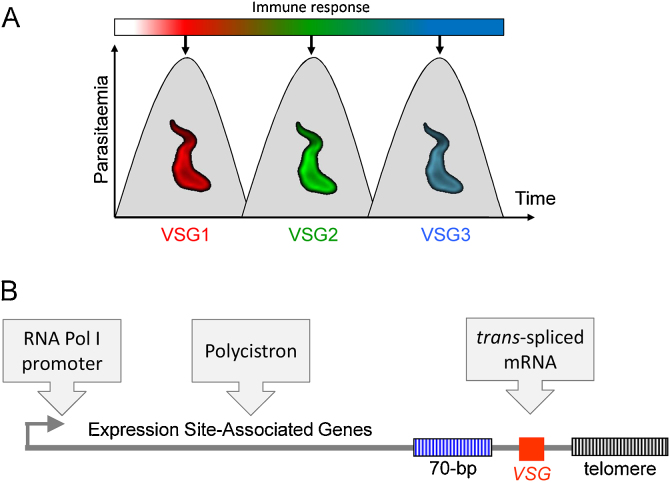
*VSG* expression and switching. (A) *VSG* switching brings about antigenic variation. Combined with successive immune responses, this can generate a relapsing parasitaemia. Natural infections are more complex than this highly simplified schematic. (B) Studies on *VSG* expression revealed some unusual features. The single expressed *VSG* was found to be flanked by distinct repetitive sequences. Three further unusual features are indicated (boxes).

Sir David Bruce had read David Livingstone's reports on the tsetse fly diseases known as nagana in cattle and sleeping sickness in humans and, while searching for the cause more than 100 years ago, reported that“ a rapidly moving object was seen lashing about among the red blood corpuscles … probably a trypanosome” [3]. Bruce also noted “the parasites come and go in the blood” and Franke & Ehrlich had deduced in 1905 that *T. brucei* acquired properties that conferred resistance to host “defensive substances”. Ronald Ross and others then enumerated the relapsing parasitaemia in patients [4], albeit treated with several different drugs during monitoring in this case. A number of parasitic infections of mammals are now known to display relapses due to the emergence of new variants that are no longer susceptible to the latest host immune response (Fig. 1A).

In the 1960s, Keith Vickerman's work using electron microscopy revealed the dense *T. brucei* coat responsible for clone-specific relapses [5]. The identification and purification of the coat proteins by George Cross followed in the 1970s [6] and then the cloning and sequencing of the corresponding cDNA in the late 1970s and early 1980s (detailed below). The VSG responsible for clone-specific immunity or antigenic variation were found to be *Variant* throughout much of their length, they were known to represent the major component of the trypanosome *Surface* coat and they were *Glycoproteins*, decorated with multiple sugar residues [6].

## 3 The variant surface glycoprotein coat

The 15 nm thick VSG coat covers the entire cell and is an essential virulence factor. Formed from approximately 10 million molecules of approximately 60 kDa, the coat represents up to 20% of total cell protein [6], facilitating the production of antisera that recognise distinct VSGs [7]. VSGs are present at the cell surface as homodimers and, despite extreme sequence divergence, display remarkably similar structures [8], partly due to a conserved arrangement of disulphide bonds. Coat exchange during a VSG-switch appears to be primarily by dilution during cell division since cells divide approximately every 6 h with shedding and turnover being relatively much slower [9]. Cytokinesis is in fact dependent upon VSG supply as demonstrated by knockdown experiments [10]. The VSG is not a transmembrane protein but is rather anchored in the membrane by glycosylphosphatidylinositol (GPI). Indeed, GPI was discovered in *T. brucei* and this yielded the first GPI structure [11] as well as a description of GPI biosynthesis [12]. GPI anchors were subsequently discovered in mammals and in other cells [13] but the biosynthetic pathways do display differences [14].

The fluid nature of the VSG coat allows for a remarkably high rate of recycling involving endocytosis at the flagellar pocket [15]. This allows the coat to be cleansed of antibodies, at low titre at least, and this requires vigorous directional cell motility mediated by the flagellum [16]. VSG coats are highly immunogenic however, so once antibody titre increases, the vast majority of parasites are eliminated and only cells with distinct VSG coats survive. As well as this variable function, the VSG coat also serves to protect less variable or even invariant surface proteins from immune effectors. Comparison of the genome sequences of *T. brucei*, *T. congolense* and *T. vivax* revealed not only how *VSG* repertoires evolved [17] but also allowed reconstruction of a surface phylome [17]. This revealed a diversity of potential non-VSG surface proteins, nutrient receptors and other ‘invariant’ surface glycoproteins. It appears that the densely packed and thick VSG coat can physically obstruct access to these proteins by conventional immunoglobulins while selectively allowing access to smaller molecules such as nutrients [18]. Nanobodies also have the potential to access the less variable epitopes usually hidden within the coat by the size exclusion limit [19].

## 4 *VSG* genes and their subtelomeric environment

Access to the first cloned *VSG* genes provided probes to explore copy number, location and diversity within trypanosome genomes, especially because the 3′ terminal regions of *VSG* mRNAs were found to be conserved [20]. It was soon recognised that the single expressed *VSG* was adjacent to a telomere, or a chromosome discontinuity that was subject to *Bal*31 exonuclease digestion [21], [22] and this strict association remains intact to date (Fig. 1B). This stimulated much interest in telomere biology in trypanosomes, which revealed the addition (and occasional large deletions) of TTAGGG/CCCTAA-repeats to growing telomeres [23], [24], [25]; the same hexameric repeats were later found at human telomeres. *T. brucei* telomeres were also found to terminate in t-loops [26] and this was followed by the identification and characterisation of telomerase reverse transcriptase, responsible for telomere growth, and other telomere-repeat-binding proteins [27].

The *VSG*-telomere association actually goes much further. Each *T. brucei* genome contains around 250 telomeres, almost all of which may be closely linked to non-transcribed *VSG* genes, with their 3′-ends closest to the telomere. Around 80% of these telomeres reside on 50–100 kbp long minichromosomes [22], [28], [29], which appear to be entirely dedicated to *VSG* archiving. Another ten or so telomeres reside on ‘intermediate’ chromosomes and the remaining 44 reside on the eleven pairs of megabase-chromosomes that comprise the diploid genome. With many additional arrays of subtelomeric *VSGs*, it is currently thought that up to 30% of an African trypanosome genome is dedicated to archiving up to 2000 *VSG* genes and gene-fragments.

Although the size of the *VSG* archive and the telomeric environment continue to present challenges for complete genome assembly and functional analyses, important insights into *VSG* gene evolution and diversification have emerged from genome sequencing [30] and the cloning and sequencing of large intact telomeric fragments [31]. Extensive hemizygous subtelomeric domains on the megabase chromosomes are dedicated to arrays of archival *VSGs* [32], meaning that many *VSGs* are present as a single copy even in a diploid genome. Most of these *VSGs* are pseudogenes in *T. brucei* (749/804 analysed) and these *VSGs* are flanked by 70-bp repeats (Fig. 1B) upstream [33] and highly conserved elements within the 3′-untranslated region (3′-UTR); both of these sequences facilitate recombination (see below). The 3′-UTR is also involved in specific stabilisation of the *VSG* mRNA at the bloodstream stage, contributing to a half-life of 4.5 h [34].

## 5 *VSG* gene expression – *trans*-splicing and polycistronic Pol I transcription

The ‘yeast to human’ view of eukaryotic diversity is very narrow so it is not surprising that several dogmas have been overturned by work on the divergent trypanosomes. Studies on *VSGs* revealed some unusual features underlying gene-expression in trypanosomatids for example (Fig. 1B). S1 nuclease protection and RNA blotting experiments revealed a spliced segment at the 5′ end of the *VSG* mRNA and reverse transcription then showed this “mini-exon” or “spliced leader” sequence to be 35 nt long [35]. Intriguingly, the same sequence was found at the 5′-end of two other *VSG* mRNAs [36]. *Cis*-splicing was initially considered to be the most likely explanation but it turned out that this same sequence was present on *all* mRNAs. In fact, discontinuous mRNA synthesis through bimolecular splicing or *trans*-splicing allows mature mRNA to be derived from precursor RNAs transcribed from two different chromosomes [37]. *Trans*-splicing was also found to operate in other trypanosomatids [38] and in nematodes.

The search for a *VSG* gene promoter extended further from the gene itself than expected. In most eukaryotes, each gene has its own promoter, while polycistrons are largely restricted to prokaryotes. Cloning and mapping upstream of an active *VSG* revealed a large expanse of imperfect 70-bp repeats [39] and then an Expression Site Associated Gene or *ESAG* [40] and then several more *ESAGs* [41] forming a polycistron. It was found that α-amanitin failed to inhibit ribosomal RNA (*rRNA*) transcription, as expected, but also *VSG* transcription [42] implicating RNA Pol I. These findings prompted a detailed analysis of RNA pol I and associated factors in *T. brucei* (see below). Polycistronic transcription was also found to operate within Pol II transcription units elsewhere in the genome [43] and this has proven to be a pervasive feature of trypanosomatid genomes. As for the *VSG* expression site promoter, the first one was eventually found around 60 kbp from the *VSG* [44] and was indeed, following mounting evidence, confirmed to recruit RNA Pol I *in vitro* [45]. Nuclear ‘run-on’ assays combined with inhibition of transcription elongation using UV exposure were instrumental in locating the *VSG* expression site promoter [46], the sequence of which was unrelated to the more conventional *rDNA* promoter [47]. The metacyclic promoters responsible for *VSG* transcription in the tsetse-fly salivary gland were also found to recruit RNA Pol I but were distinct from the bloodstream promoters and in this case were located only a short distance upstream of the telomeric *VSGs* [48]; these are among only a few monocistronic transcription units in trypanosomatids.

The result of all this work revealed a somewhat surprising situation whereby RNA Pol I and RNA Pol II are required to produce mature *VSG* expression site transcripts; transcription of the spliced leader sequence was confirmed to be RNA Pol II dependent [49]. Further surprises were in store as assessment of other potential Pol II promoters in *T. brucei* or in other trypanosomatids failed to yield any further conventional examples. Rather, around sixty RNA Pol II ‘transcription initiation sites’ appear to depend upon a particular chromatin structure [50] that is not readily reconstituted on reporter constructs.

Fifteen copies of the highly conserved subtelomeric *VSG* expression sites active in the bloodstream have now been identified, cloned by recombination in yeast and sequenced from the most widely studies *T. brucei* strain [31]. Present on the diploid and intermediate-chromosomes, these contain intact promoters and are competent transcription units but are typically reversibly repressed due to monoallelic *VSG* expression control. Many *ESAGs* found within these polycistronic units remain to be characterised but the evidence so far points to roles in host-parasite interactions. For example, a novel heterodimeric transferrin receptor, encoded by *ESAG6* and *ESAG7* and related to the *N*-terminal domain of the VSG, has the capacity to bind transferrin from different hosts with different affinities [51]. A human serum resistance associated gene, or *SRA*, found in *T. brucei rhodesiense*, also resembles a *VSG* and is also an *ESAG* [52] while another gene resembling a *VSG*, known as *TgsGP*, confers human-serum resistance to *T. brucei gambiense* [53]. *ESAG4* genes are unrelated to *VSG*s, but also mediate host-parasite interactions. These genes encode adenylate cyclases which are released by lysed trypanosomes and inhibit the innate immune response [54]. Clearly, *VSGs* and their associated genes have been central to the ‘arms-race’ operating and evolving at the host-parasite interface. The vast reservoir available has allowed *VSG* genes to be co-opted to functions beyond classical antigenic variation. The relationships among these proteins could equally reflect an evolutionary origin of VSGs from ancient surface receptors.

## 6 Antigenic variation by *VSG* gene rearrangement

Subtelomeres are recombinogenic hotspots, plastic regions of genomes enriched in gene families that are most commonly involved in adaptation to different environments. Like many other cell types, African trypanosomes appear to have exploited these properties, in this case for the massive expansion and evolution of the *VSG* family and also for the *ESAGs* [55]. Antigenic variation in *T. brucei* involves switching to expression of a distinct *VSG* so the availability of *VSG* cDNA clones allowed researchers to look for changes associated with the activation and inactivation of those *VSGs*. Early analyses using Southern blotting [56], [57], northern blotting [58], DNAse I digestion [59] or DNA sequencing [60] revealed switching by duplicative transposition of a non-telomeric ‘basic copy’ *VSG* and replacement of the old *VSG* by the new ‘expression-linked copy’ at a single transcribed locus. Silent telomeric *VSG* cassettes appeared to be copied all the way to the end of the chromosome [61], [62] by a mechanism now known as ‘break-induced replication’ (BIR).

**Fig. 2:**
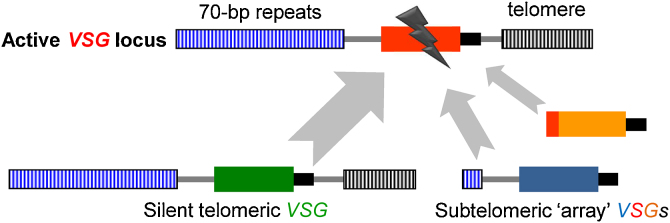
DNA recombination is central to *VSG* switching. The active subtelomeric *VSG* locus is prone to spontaneous DNA breaks. Three classes of repair templates are shown with *VSGs* represented as coloured boxes; those sharing more (flanking) homology with the active site are used more frequently (grey arrows). The homologous sequences indicated are the 70-bp repeats (blue stripes), the telomeric repeats (black stripes), the *VSG* 3′-UTRs (thick black bars) and a portion of the *VSG* coding sequence (red). A break in or around the active *VSG* is followed by DNA resection extending towards the 70-bp repeats, often initiating recombination in this region.

Subsequent analyses revealed a range of variations on the recombination theme and DNA homology emerged as the key driver of these *VSG* rearrangements [63] stimulating further studies on a range of *T. brucei* DNA-repair factors. Most *VSGs* are flanked upstream by common 70-bp repeats and even non-telomeric genes share sequences at the 3′-end [64], sometimes within the region encoding the VSG *C*-terminal domain and almost always in the conserved *VSG* 3′-UTR (Fig. 2). Replacement of larger parts of the expression site or non-duplicative telomere exchange [65] can bring about a switch but these events appear to be relatively rare and even actively suppressed [66]. Translocation to the active site is preferentially initiated by the long tracts of 70-bp repeats found upstream of the active *VSG* [67], [68] so the probability of being ‘selected’ as a template for repair depends upon these and other sequences shared with the active *VSG* locus [69]. Indeed, 70-bp repeat recombination is primarily responsible for the high-efficiency duplication of telomeric *VSGs* [61], [62], which can employ the BIR mechanism, while chromosome-internal *VSGs* must use a ‘gene-conversion’ mechanism, similar to BIR but with a second recombination junction, most often within the *VSG* 3′-UTR [39], [70]. Conversion of even shorter *VSG* segments can generate ‘mosaic’ *VSGs* that, because of their lower-frequency emergence, become increasingly important for parasite persistence in a chronic infection [64], [71], [72], [73], as immunity to more-frequently activated VSGs builds [67].

This understanding of the shared sequences that drive recombination can now explain why the expression of *VSGs* is ‘semi-ordered’ or somewhat predictable. Any order though will be isolate-dependent, less predictable as the infection progresses and will ultimately be highly dynamic and variable in different infections and also from one epidemic to the next, meaning that herd-immunity is unlikely to be achieved. The potential *VSG* repertoire is ultimately larger than a single genomic repertoire due to segmental gene conversion and inter-strain mating in the tsetse fly [74]. This genome plasticity also impacts the *VSGs* expressed in the tsetse fly salivary gland and presents stark challenges in terms of any vaccine strategy that targets VSGs.

*VSG* recombination does not appear to be naturally triggered by a site-specific nuclease but rather appears to depend upon the inherent instability of subtelomeres [75], [76]. Breaks were shown to arise naturally at telomeric *VSG* loci, probably due to replication fork collapse, and an artificial, meganuclease-induced DNA break at the active site can trigger a switch [75], [77]. These breaks initiate DNA resection producing ssDNA and triggering a homology search. Once a suitable template is found, DNA can be copied from the template to repair the lesion [76]. Non-homologous DNA repair does not appear to operate in *T. brucei*, placing an emphasis on RAD51-dependent homologous recombination [63], [78]. This has had a major impact on our ability to manipulate the *T. brucei* genome and also makes an important contribution to *VSG* gene rearrangements. There is an alternative form of microhomology-mediated end-joining, however. This end-joining is RAD51-independent and may be particularly effective within 70-bp repeats, thereby making a substantial contribution to the duplicative transposition of *VSG*s [76].

Nuclear positioning and the chromatin environment of *VSG*s may be important for *VSG* recombination [77]. In the bloodstream form, telomeres [79], active [80] and silent *VSG* expression sites [81] are distributed throughout the nuclear space rather than sequestered at the periphery and this may facilitate homology searching during DNA repair. Notably, the active *VSG* expression site specifically migrates to the nuclear periphery during differentiation to the insect stage [82].

Switching occurs in only approximately 0.001% of cells per cell division cycle in experimental *in vitro* culture or during frequent syringe passage but appears to be much higher naturally; switch rate returns to >0.2% following transmission through flies [83] and also apparently increases during growth *in vivo* in mammals [68], [84]. This transition is not understood but could involve the acquisition of a hyper-labile or hyper-recombinogenic state at the active *VSG* locus [85]. Rapid switching also operates in metacyclic cells obtained from tsetse-fly salivary glands [86] but in this case, switching does not involve recombination [87].

The vast and dynamic *VSG* gene family, the large number of subtelomeres in *T. brucei* and the incomplete sequence coverage currently available for these regions presents challenges but current techniques should now allow some longstanding questions to be addressed. For example, where in the genome are often entirely pseudogene-derived mosaic-*VSGs* assembled and presumably selected for? Do non-templated *VSG* mutations [88] naturally contribute to immune evasion? Are novel *VSGs* typically preserved or permanently lost once successfully used for immune evasion? In terms of this last question, the presence of intact *VSGs* on minichromosomes [29] could reflect an effective archiving mechanism for novel *VSGs*. It will also be of interest to determine whether *VSG* recombination requires or exploits dedicated or specifically modified DNA repair factors, such as BRCA2 with an expanded set of RAD51-interacting repeats [89].

## 7 Antigenic variation by *VSG* transcription (in)activation – allelic exclusion

**Fig. 3:**
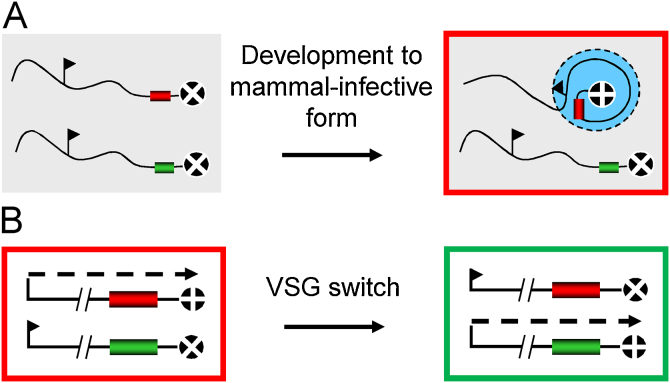
*VSG* allelic exclusion is not yet understood. (A) Only one of the telomeric *VSGs* (red and green) is active and only in the mammalian stages. This associates with a focus of extranucleolar RNA Pol I and other factors (light blue) and produces the VSG coat (outer red box). The ‘+’ and ‘x’ symbols indicate the active and silent telomeres, respectively. (B) Transcription can occasionally switch from one telomeric *VSG* to another; a coordinated epigenetic switch maintains allelic exclusion. The dashed arrow indicates transcription. Other details as in A.

Studies on *T. brucei* clones with switched VSGs also revealed that some switching events were not associated with duplicative *VSG* transposition [90]. Pulsed-field gel-electrophoresis, combined with Southern blotting, which had also facilitated ‘mapping’ of the *VSG* rearrangements described above, provided confirmation of coordinated on and off switching, known as ‘*in situ*’ switching [28]. This showed that certain telomeric *VSGs* were reversibly active or repressed and could be subject to transcriptional switching as well as recombination [61], [91]. The *in situ* switches are classical epigenetic switches since they occur without changes in the DNA sequence [44]. Thus, although gene expression control in trypanosomatids is primarily post-transcriptional, *VSG* exclusion represents a prominent exception (Fig. 3). Although simultaneous expression of two VSGs from distinct telomeric sites has been reported in *T. equiperdum* [92], VSG double-expressors arose rarely and were unstable in *T. brucei* [93]. Thus, *VSG* allelic exclusion is generally strictly maintained.

The mechanism that distinguishes among almost identical *VSG* expression sites in the bloodstream form suppresses both transcription initiation [94] and elongation [95] and this results in a remarkable 10,000-fold abundance differential among active and silent *VSG* mRNAs [96]. Despite the distinct sequences of *rDNA* promoters, these promoters, inserted at *VSG* expression sites, adopt the transcriptional status of that site [97], [98]. A second *VSG* inserted at an active *VSG* expression site is similarly also active [99], [100]. In contrast to this locus-specific control in the bloodstream form, it seems that *VSG* promoter-specific elements, distinct from *rDNA* promoters, allow selective down-regulation of all *VSG* transcription during differentiation to the insect-stage [101].

A novel DNA base, the hydroxylated and glucosylated derivative of thymidine or base-J [102], was first discovered at silent *VSG* loci. Evidence of J-base came from blocked cleavage by certain restriction enzymes such as *Pst*I as revealed on Southern-blots. Base-J is notably absent from insect-stage *T. brucei* but, although implicated for many years, no role in antigenic variation has been demonstrated [103]. What is established is that J-base is required for RNA Pol II transcription termination in *L. major* [104] so it remains possible that J-base also presents a barrier to certain RNA polymerases or DNA polymerases in *T. brucei*, potentially impacting transcription or DNA recombination and repair, respectively.

Transcription of the single active *VSG* by RNA Pol I, rather than RNA Pol II, suggested a potential ‘privileged domain’ model for activation based on association with the nucleolus. As it turned out, active *VSG* transcripts and the active *VSG* locus were found to be extranucleolar [81]. Like the nucleolus though, this region, known as the expression-site body, is associated with an accumulation of RNA Pol I [80], a trypanosomatid-specific transcription initiation complex known as class I transcription factor A [105] and also a pol I-associated high-mobility group factor [106].

It remains likely that the telomeric environment is important for *VSG* allelic exclusion as well as for *VSG* recombination. There is evidence of a role for the telomere-binding protein, RAP1, in *VSG* silencing [96] and this silencing is also dependent upon chromatin structure [107], [108]; since this topic is covered in a recent review [77] it is not covered in any detail here. Briefly, the histones, histone chaperones, chromatin remodelers, chromatin modifiers, cohesins and nuclear lamina, as well as other chromatin-associated factors, contribute to repression. DOT1B in particular, has a major role in establishing the silent state during *in situ* switching [109]. Thus, transcription is clearly repressed or attenuated at telomeric *VSG* loci. Understanding allelic exclusion will remain challenging until we know more about the selection of a single *VSG* for expression and how this is coordinated with the silencing of all other *VSGs*.

## 8 Concluding remarks

The abundance of the active VSG, quite stable yet reversible *VSG* repression and the ease of genetic manipulation and cell culture mean that *T. brucei* provides a highly tractable experimental system for the study of monoallelic expression and antigenic variation. There has been tremendous progress in our understanding in this area, how the *VSGs* are organised and expressed and how expression is switched, through recombination in particular. An ancient and ongoing ‘arms race’ between host immunity and parasite immune evasion has been illuminated through studies on VSGs. The set of monocistronic VSGs first expressed in the tsetse-fly salivary gland facilitates the establishment of a mammalian infection following a blood-meal. The multiplicity of telomeric *VSGs* with alternative collections of *ESAGs* may then provide an opportunity to select the optimal expression site for effective nutrition and growth in distinct host environments [51]. Recombination can then allow for *VSG* switching compatible with continued expression of a favoured set of *ESAGs*. The vast reservoir of *VSG* genes allows the presentation of constantly changing epitopes at the cell surface to counter the hosts’ capacity for adaptive immunity.

Access to genome sequence data changed the research landscape, allowing easy access to a vast number of *VSG* sequences, factors involved in transcription, telomere-binding, recombination and repair and chromatin-based control. There has been an inevitable focus on factors related to those with known functions in other eukaryotes, however, meaning that a lot of territory still remains uncharted in trypanosomatid research. Recent technical advances in areas such as forward genetics [110], proteomics [111], improved access to *T. brucei* developmental stages in *in vitro* culture [112] and new and improved technologies to come, will surely now help to deliver answers to some of the outstanding questions.
